# Antimicrobial Activity of Isothiocyanates from Cruciferous Plants against Methicillin-Resistant *Staphylococcus aureus* (MRSA)

**DOI:** 10.3390/ijms151119552

**Published:** 2014-10-28

**Authors:** Carla Dias, Alfredo Aires, Maria José Saavedra

**Affiliations:** 1 Animal and Veterinary Research Centre, CECAV, University of Trás-os-Montes and Alto Douro, UTAD, Quinta de Prados, Vila Real 5000801, Portugal; E-Mails: cdias@utad.pt (C.D.); saavedra@utad.pt (M.J.S.); 2 Centre for the Research and Technology of Agro-Environmental and Biological Sciences, CITAB, University of Trás-os-Montes and Alto Douro, UTAD, Quinta de Prados, Vila Real 5000801, Portugal

**Keywords:** chemical structure, bioactivity, antibiotic resistance, bioactive compounds

## Abstract

Purified isothiocyanates from cruciferous plants (Brassicacea, Syn. Cruciferae) plants were evaluated against 15 isolates of methicillin-resistant *S. aureus* isolated from diabetic foot-ulcer patients aiming the study of the potential usage of allyl-isothiocyanate, benzyl-isothiocyanate and 2-phenylethyl-isothiocyanate against this important bacteria. Disc diffusion and minimum inhibitory concentration methods were used to access the antimicrobial activity. The index (Ia) and rate (Ra) of the antibacterial activity for each compound were calculated. The results showed a highly dose-dependent compound and chemical structure antibacterial effectiveness. The results showed a strong relation between the chemical structure of isothiocyanates and its antibacterial effectiveness. The benzyl-isothiocyanate was the most effective with a minimum inhibitory concentration varying between 2.9 and 110 µg· mL^−1^ with an antibacterial activity rate up to 87%. Moreover, their antibacterial activity was mainly bactericidal. This study provides scientific evidence that isothiocyanates have an interesting biological value and must be considered as an important tool to be used against MRSA.

## 1. Introduction

**Figure 1:**
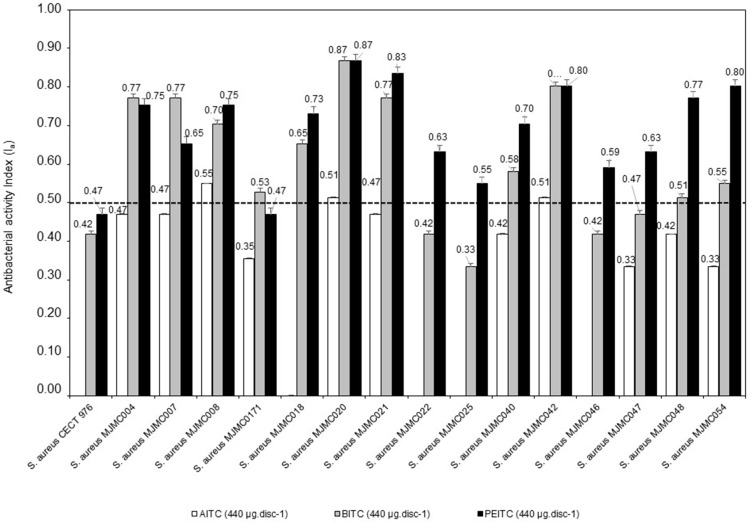
Average levels of the antibacterial index (Ia) for each MRSA isolate and ITCs. Allyl-isothiocyanate (AITC); Benzyl-isothiocyanate (BITC); 2-Phenylethyl-isothiocyanate (PEITC).

The growing incidence of infectious diseases due to development of increasing antibiotic resistant pathogens justify the attempts to search for new antimicrobial agents as well as compounds that are capable of inhibiting resistance bacterial mechanisms to classical drugs. The continuous use of the same class and type of antimicrobial compounds seems to contribute to a significant increase in resistant bacteria, particularly resistant Gram-positive organisms. Most relevant research papers are concerned with the increase in resistant *Streptococcus pneumoniae*, methicillin-resistant *Staphylococcus aureus* (MRSA) and vancomycin-resistant enterococci (VRE) over recent years [1]. *Staphylococcus aureus* causes purulent skin and soft tissue infections that frequently reoccur. *S. aureus* is considered one of the main pathogens causing nosocomial infections and the occurrence of antibiotic resistant strains of *S. aureus* within hospitalized patients is still recurrent [2,3] and can endorse invasive diseases which are among the most significant causes of infectious disease mortality in both developed and developing countries, particularly in those where the economical investment in new drugs, therapies and clinical approaches is often extremely limited. Moreover, human or animal infections with *S. aureus* do not elicit protective immunity against staphylococcal diseases [3]. MRSA infections in humans have been associated with excess morbidity and increased length of hospitalization [4]. Despite the discovery of new drugs for MRSA and different non-drugs interventions, outbreaks of MRSA infections often appear in hospitals, clinics, nursing and elderly homes. Moreover, recently, MRSA have been reported in animals and people who work with animals in a persistent way [5]. To overcome this problem, one approach could be the improvement of the current drugs to overcome drug-induced resistance (caused by mutations of the targets) or to identify new classes or types of antibacterial compounds. Plants could be an interesting choice since they have an extensive range of compounds with important biological properties including antimicrobial activity. Among these natural compounds are the isothiocyanates. Isothiocyanates (ITCs) are an important class of compounds derived by enzymatic hydrolysis (*myrosinase*, thioglucose glucohydrolase (EC 3.2.3.1.)) from aliphatic, indole and aromatic glucosinolates [6] (Figure 1). Glucosinolates (GLs) are sulfur-containing compounds largely present in Brassicaceae (Syn. Cruciferae) plants. According to their chemical structure they are grouped in three mains classes namely: aliphatics, indoles and aromatics, which after enzymatic hydrolysis originate among others: aliphatics, indoles and aromatics ITCs [7] (Figure 1). The GLs hydrolysis products have been reported as having important biological effects and being responsible for several biological effects in humans, particularly protection against chemical carcinogens [8,9] and acting as major inhibitors of microbial activity [10].

Research studies have shown that ITCs exhibit also a biocide activity against microorganisms, as well as insects and pests. It has been demonstrated that allyl ITC (AITC) from sinigrin (2-propenyl glucosinolate) and 2-phenylethyl ITC (PEITC) effectively inhibits a variety of pathogenic microorganisms such as *Salmonella Montevideo*, *Escherichia coli* O157:H7, *Listeria monocytogenes* Scott A, *Vibrio parahaemolyticus*, *Bacillus cereus*, *Bifidobacterium*, *Clostridium and Lactobacillus* [11,12,13,14]. Regardless these results, no major studies have been conducted with MRSA isolates and the data reach does not yet allow clear deductions about the relative efficiency of ITCs against these bacteria, particularly about their structure-function relationship, their action mode or their compared spectra of activity. Therefore, to increment the scientific knowledge about their potential against MRSA we present this *in vitro* study in which we evaluate the antibacterial activity of allyl-isothiocyanate (AITC), benzyl-isothiocyanate (BITC) and 2-phenylethyl-isothiocyanate (PEITC) (Table 1), largely present in cruciferous plants [15,16,17,18,19] and often reported as having nematicidal, antifungal and antiviral properties as antimicrobial agent against plant pathogens [6,15,16,17,18,19].

**Table 1:** Inhibitory diameter zone in mm for Isothiocyanates (ITCs) against Methicillin-resistant Staphylococcus Aureus (MRSA) isolates.^1,2,3,4^

Bacteria Isolates	AITC (440 µg·disc^−1^)	BITC (440 µg·disc^−1^)	PEITC (440 µg·disc^−1^)	Vancomycin (30 µg·disc^−1^)
*S. aureus CECT 976* (reference strain)	-	10.3 ± 0.6a	11.3 ± 0.6a	15.7 ± 0.6b
*S. aureus MJMC004*	11.3 ± 0.6a	26.3 ± 0.6d	24.3 ± 0.6c	15.7 ± 0.0b
*S. aureus MJMC007*	11.3 ± 0.9a	26.3 ± 0.6d	17.3 ± 0.9c	14.7 ± 0.0b
*S. aureus MJMC008*	13.3 ± 0.6a	20.3 ± 0.6c	24.3 ± 0.6d	15.7 ± 0.0b
*S. aureus MJMC0171*	9.3 ± 0.6a	12.7 ± 0.6c	11.3 ± 0.6b	14.7 ± 0.0d
*S. aureus MJMC018*	-	17.3 ± 0.6b	22.3 ± 0.6c	16.7 ± 0.0a
*S. aureus MJMC020*	12.3 ± 0.6a	45.3 ± 1.6c	45.3± 1.2c	15.7 ± 0.0b
*S. aureus MJMC021*	11.3 ± 0.6a	26.3 ± 0.6c	36.3 ± 1.2d	15.7 ± 0.0b
*S. aureus MJMC022*	-	10.3 ± 0.1a	16.3 ± 0.6b	15.7 ± 0.0b
*S. aureus MJMC025*	-	9.0 ± 0.1a	13.3 ± 0.6b	13.7 ± 0.0b
*S. aureus MJMC040*	10.3 ± 0.1a	14.3 ± 0.6b	20.3 ± 0.6c	15.7 ± 0.0b
*S. aureus MJMC042*	12.3 ± 0.1a	30.3 ± 1.0c	30.3 ± 1.2c	16.7 ± 0.0b
*S. aureus MJMC046*	-	10.3 ± 0.6a	14.7 ± 0.6b	16.7 ± 0.0c
*S. aureus MJMC047*	9.0 ± 0.6a	11.3 ± 0.6b	16.3 ± 0.6c	15.7 ± 0.0c
*S. aureus MJMC048*	10.3 ± 0.1a	12.3 ± 0.6b	26.3 ± 0.6d	15.7 ± 0.0c
*S. aureus MJMC054*	9.0 ± 0.1a	13.3 ± 0.6b	30.3 ± 1.2c	15.7 ± 0.0b

^1^ (-) No bacterial effect; ^2^ AITC-Allylisothiocyanate; BITC-Benzylisothiocyanate; PEITC-2-Phenylethylisothiocyanate; ^3^ Values presented are means of triplicate determinations ± standard deviation (SD); ^4^ Different letter in same row mean different value at significance level of *p* < 0.05 by *t*-student test.

## 2. Results and Discussion

### 2.1. Chemical Structure of Isothiocyanates.

The chemicals AITC, BITC, and 2-PEITC (95%, 98% and 99% of purity, respectively) were purified from cruciferous plants. They present different chemical structure, AITC being an aliphatic compound and BITC and PEITC both being aromatic with a benzene ring. This structural difference reflects not only the lipophilic and hydrophilic properties but also their antimicrobial, antioxidant and anticancer potential [6,10]. According to some authors [14], compounds composed by short carbon chains can be more aggressive against bacteria survival structures due to higher efficiency and stability in linkage with bacteria structures. Therefore, is expected that compounds with shorter chains have more antibacterial activity.

### 2.2. Antimicrobial Activity of ITCs against MRSA

Several studies have shown that MRSA strains are highly resistant to antibiotics [14,20,21,22]. Thus, research for new and effective antimicrobial agents and/or alternative approaches to the conventional use of antibiotics are urgently needed. 

The antimicrobial activity of the selected isothiocyanates with disc diffusing assay is presented in Table 1. The zone of inhibition detected were in general higher for BITC and PEITC and lower for AITC. For AITC the zone of inhibition values were always lower than 15 mm, whilst for the other two ITCs the halos varied between 9.0 and 45.3 mm for BITC and between 11.3 and 45.3 mm for PEITC. The antibacterial activity index (Ia) (Figure 1) showed that in 15 MRSA isolates, two were both highly susceptible to BITC and PEITC (13% of isolates tested), two isolates were more susceptible to BITC (13%) and 10 isolates more susceptible to PEITC (63%). Thus, their antibacterial rate (Ra) effectiveness were variable but, for the majority of the isolates, the Ra were always higher than 55%. The maximum was 87% for both BITC and PEITC. For AITC, the Ia were always lower than 0.50 (Figure 1) and thus their Ra effectiveness was lower than 50%.

The results with minimum inhibitory concentrations (MIC) assay are presented in the Table 2. In general the AITC always presented the higher MIC values and thus lower antimicrobial activity, whilst BITC and PEITC presented the lowest MIC. Therefore, these isothiocyanates showed the highest antimicrobial activity, confirming the previous results for disc diffusion assay. The average MIC values for BITC were significantly different (*p* < 0.05) from PEITC. The AITC and PEITC were essentially bacteriostatic whilst BITC was bactericidal in MRSA 11 isolates (representing 69% of isolates tested) (Table 2). Based on this BITC is more effective in suppressing MRSA strains than PEITC.

**Table 2:** Minimum Inhibitory concentration (MIC) (µg·mL^−^^1^) and bacterial effect of ITCs against MRSA isolates.^1,2^

Bacteria Isolates	AITC		BITC		PEITC	
	MIC	Main Effect	MIC	Main Effect	MIC	Main Effect
*S. aureus* CECT 976 (reference strain)	110.0 ± 0.0b	bacteriostatic	55.7 ± 0.0a	bactericidal	110.0 ± 0.0b	bacteriostatic
*S. aureus MJMC004*	110.0 ± 0.0c	bacteriostatic	2.9 ± 0.0a	bacteriostatic	55.7 ± 0.0b	bacteriostatic
*S. aureus MJMC007*	36.7 ± 0.01b	bacteriostatic	4.4 ± 0.0a	bacteriostatic	7.3 ± 0.0a	bacteriostatic
*S. aureus MJMC008*	220.0 ± 0.0c	bactericidal	23.5 ± 0.01a	bacteriostatic	110.0 ± 0.0b	bacteriostatic
*S. aureus MJMC0171*	110.0 ± 0.0b	bactericidal	55.7 ± 0.0a	bactericidal	110.0 ± 0.0b	bactericidal
*S. aureus MJMC018*	220.0 ± 0.0b	bacteriostatic	110.0 ± 0.0a	bactericidal	183.3 ± 0.4b	bactericidal
*S. aureus MJMC020*	110.0 ± 0.0a	bactericidal	110.0 ± 0.0a	bactericidal	110.0 ± 0.0a	bacteriostatic
*S. aureus MJMC021*	110.0 ± 0.0b	bactericidal	55.7 ± 0.0a	bactericidal	110.0 ± 0.0b	bacteriostatic
*S. aureus MJMC022*	220.0 ± 0.0c	bacteriostatic	110.0 ± 0.0a	bactericidal	146.7 ± 0.04b	bacteriostatic
*S. aureus MJMC025*	110.0 ± 0.0a	bacteriostatic	110.0 ± 0.0a	bactericidal	110.0 ± 0.0a	bacteriostatic
*S. aureus MJMC040*	27.9 ± 0.0a	bacteriostatic	<7.3 ± 0.0	bactericidal	55.7 ± 0.0b	bacteriostatic
*S. aureus MJMC042*	110.0 ± 0.0b	bacteriostatic	13.2 ± 0.0a	bactericidal	146.7 ± 36.7b	bacteriostatic
*S. aureus MJMC046*	55.7 ± 0.0c	bacteriostatic	7.3 ± 0.0a	bactericidal	27.9 ± 0.0b	bacteriostatic
*S. aureus MJMC047*	220.0 ± 0.0b	bactericidal	7.3 ± 0.0a	bactericidal	<7.3 ± 0.0	bactericidal
*S. aureus MJMC048*	110.0 ± 0.0b	bacteriostatic	55.7 ± 0.0a	bacteriostatic	110.0 ± 0.0b	bacteriostatic
*S. aureus MJMC054*	110.0 ± 0.0b	bacteriostatic	110.0 ± 0.0b	bacteriostatic	110.0 ± 0.0b	bacteriostatic

^1^ AITC-Allylisothiocyanate; BITC-Benzylisothiocyanate; PEITC-2-Phenylethylisothiocyanate; ^2^ Values presented are means of triplicate determinations ± standard deviation (SD); ^3^ Different letter in same row mean different value at significance level of *p* < 0.05 by *t*-student test.

Our results showed an interesting antimicrobial potential isothiocyanates against MRSA isolates. The *in vitro* screening disc diffusion assay revealed that selected isothiocyanates are interesting antimicrobial compounds even if the zone of inhibition are not detected visually. This seems contradictory but as the results showed by the disc diffusion assay, MIC values and antibacterial effect assays (Table 2) for the isolate MJMC007, we can have a situation in which a specific compound has antibacterial activity but without the development of an inhibition halo. Thus, the antibacterial screening with disc diffusion assay by itself alone may not be the best method to evaluate the antibacterial effectiveness of any compound, although it is still one of the most used methods. Another important factor behind the different antibacterial activity effectiveness exhibited by the isothiocyanates is their specific chemical structure. Our results showed that variations in chemical structure imply different grade and type of antibacterial activity. In fact, based in our results it seems that aromatic rings (as exhibited by BITC and PEITC) are highly fundamental for antibacterial effectiveness. In addition, short carbon chains with oxygens can give an extra aggressiveness to compounds as shown by BITC. In opposition, compounds with longer carbon chains (PEITC) and/or without aromatic rings such as AITC, are less effective against bacteria. Actually, it is well accepted that sulfhydryl groups easily bound with specific enzymes fundamental to microbial growth and survival [14]. The linkage of this groups to these enzymes restricts their enzymatic capacity causing reduction in the cellular levels of important thiol groups and then leading to formation of oxygen and other free-radicals [23] and thus reducing the viability of bacterial cells. The BITC due to its short chain can have more efficiency in stopping or killing bacteria. Moreover, its indole structural formula might interfere more efficiently in the peptidoglycan biosynthesis, avoiding the assembly and protein synthesis which is fundamental for bacteria survival. Also, based on both lipophilic and electrophilic properties, BITC can be more capable of moving throughout bacteria structures interfering with bacterial redox system and consequently stopping the ability of bacteria to maintain its internal potential. All these properties together can give BITC more antibacterial efficacy. This result is important since only a few studies have been published about the effectiveness of isothiocyanates against pathogenic bacteria, and even fewer about its effectiveness against MRSA.

Although different studies have addressed some possible adverse effects of isothiocyanates and particularly BITC on human health, the *in vivo* tolerance is very high when compared to the *in vitro* effects on cell-lines [24,25] indicating that BITC is a detoxifying molecule in living organisms as reported. Thus, it is unlikely that BITC can cause toxicity when used in wounds or ulcers in diabetic patients, although the LC50/LD50 values must be respected. Thus, plants with higher content of ITCs, such as mustards, broccoli, cabbage, turnips, watercress or other native ethnobotanical cruciferous plants must be studied in order to assess their potential as a natural source of these or even other types of ITCs.

## 3. Experimental Section

### 3.1. Methicillin-Resistant Staphylococcus Aureus (MRSA) Isolates

Sixteen clinical isolates MRSA, including one MRSA standard strain CECT (S. aureus CECT 976) were used in this study (Table 1 and Table 2). Except the standard strain, all isolates were isolated and identified by Dr. João Gaspar and Dr. Eulália Carvalho from Chirurgic department and Clinical pathology laboratory (Hospital of Trás-os-Montes and Alto Douro, Vila Real, Portugal) from skin and wounds of human diabetes patients. All cultures were maintained at −80 °C in brain heart infusion (BHI) broth (Bio-Rad, Marnes-La-Coquette, France) with 30% glycerol until experimental use. Before *in vitro* assay, the isolates were cultured for 18 h at 30 °C in BHI broth. Cultures were then diluted in fresh BHI broth and adjusted to a final concentration of 105 CFU·mL^−1^. Different antibiotics like Methicillin, Vancomycin, Chloramphenicol, Erythromycin, Ampicillin and Penicillin (at 10 micro gram concentration) were used (Prof. Maria José). In the end, 16 isolates MRSA were used in this study.

### 3.2. Isothiocyanates (ITCs)

The AITC, BITC, and 2-PEITC (95%, 98% and 99% of purity, respectively). The solutions of ITCs at 440 µg·disc^−1^ were prepared in 10% dimethyl sulfoxide (DMSO) (Sigma-Aldrich, Taufkirchen, Germany). Working solutions of each ITC were prepared previously at each assay and stored at −20 °C for no more than 1 week.

### 3.3. Antimicrobial Activity by Disc Diffusion Method

The ITCs were first screened against MRSA isolates by in vitro disc diffusion method according the Clinical and Laboratory Standards Institute [26]. Isolated colonies from pure strains were transferred from the BHI solid medium grown overnight and inoculated into 4.0 mL of 0.9% NaCl solution. From these stocks, suspensions were prepared by adjusting the turbidity to 0.5 McFarland standard units. A loop of bacteria from suspension was spread with a sterile cotton swab into Petri dishes (90 mm of diameter) containing 20 mL of Mueller-Hinton Agar (Oxoid, Basingstoke-Hampshire, UK) and a sterile filter paper discs (6 mm in diameter) (Oxoid, Basingstoke-Hampshire, UK) impregnated with 15 µL of ITCs were then placed on the agar plate. Then the plates were incubated overnight at 37 °C. A negative control (15 µL of solvent, DMSO) and positive control (commercial antibiotic of vancomycin in discs (VA, 30 µg, Oxoid, Basingstoke-Hampshire, UK) were used. At the end of the incubation, the diameter of inhibition zones were measured on the plates with a ruler and recorded in mm. All tests were performed in triplicate and the antibacterial activity was expressed as the mean of inhibition zone diameters. The antibacterial activity index (Ia), which makes it possible to compare efficiency of different compounds with same concentration was calculated with the following formula: Ia = (A1 − A0)/A1, where A1 is de diameter of inhibition zone of compound tested, A0 is the diameter of sterile paper disc. This value converted into a percentage gives the antibacterial activity rate (Ra).

### 3.4. Antimicrobial Activity Minimum Inhibitory Concentration (MIC)

For the MIC assay a modified resazurin microtitre-plate assay [27] was used. Briefly, 100 μL of each ITC (440 µg·well^−1^) and standard antibiotic in 10% DMSO solution was pipetted into the first row of the 96 well microplates. To all other wells, 100 μL of nutrient broth was added. Two fold serial dilutions were performed using a multichannel pipette such that each well had 100 μL of the test material in serially descending concentrations. Twenty microliters of bacterial suspension was added to each well to achieve a concentration of 5 × 105 cfu·mL^−1^. Finally, 20 μL of resazurin indicator solution (270 mg of pure resazurin in 40 mL of sterile bi-distilled water) were added to each well. At the end, the microplate was covered with cling film to avoid dehydration of bacteria. Each microplate had a set of controls: a column with VA as positive control; a column with DMSO solution as a negative control; a column with all components except bacterial suspension (contamination control) and column with bacteria suspension (bacteria growth control). The plates were prepared in triplicate and incubated at 37 °C for 24 h. The color change was assessed visually. The bacterial growth was indicated by color changes from purple to pink (or colorless) and the lowest concentration at which color change occurred was considered MIC value. Then, aliquots from each MIC wells were transferred to agar plates and incubated overnight at 37 °C. The concentrations that did not permitted any visible growth (no colonies) were considered as being bactericidal effect and those with visible growth were considered as bacteriostatic. Each assay was performed in three replicates and the mean value was recorded with standard deviation.

### 3.5. Data Analysis

All experiments were conducted in triplicate and statistical analyses were conducted using analysis of variance (ANOVA). The results were expressed as means of triplicate determinations ± standard deviation (SD). The confidence limits used in this study were based on 95% (*p* < 0.05). The *t*-student test was used to separate means at *p* < 0.05. The software SPSS v.17 (SPSS-IBM, Orchard Road-Armonk, New York, NY, USA) was used for statistical analysis.

## 4. Conclusions

Based on our results, we can conclude that cruciferous plants such as mustards, cress, watercress and turnips, can play an important role as natural source of BITC and PEITC, both with strong antimicrobial activity against MRSA. Thus, these specific compounds can be used in pharmacological formulations, alone or in combination with traditional antibiotics against MRSA. Synergistic studies with antibiotics or other natural compounds with recognized antibacterial activity should be studied experimentally in order to obtain more information, including toxicity.

## References

[1] Schoenfeld E.M., McKay M.P. (2010). Mastitis and methicillin-resistant *Staphylococcus aureus* (MRSA): The calm before the storm?. J. Emerg. Med..

[2] Kim H.K., Thammavongsa V., Schneewind O., Missiakas D. (2012). Recurrent infections and immune evasion strategies of *Staphylococcus aureus*. Curr. Opin. Microbiol..

[3] Montgomery C.P., Daniels M., Zhao F., Alegre M.L., Chong A.S., Daum R.S. (2014). Protective immunity against recurrent *Staphylococcus aureus* skin infection requires antibody and interleukin-17A. Infect. Immun..

[4] Libert M., Elkholti M., Massaut J., Karmali R., Maskart G., Cherifi S. (2008). Risk factors for methicillin-resistantce and outcome of *Staphylococcus aureus* blood stream infection in a Belgian university hospital. J. Hosp. Infect..

[5] Anderson M.E.C., Lefebvere S.L., Weese S.J. (2008). Evaluation of prevalence and factors for methicillin-resistant *Staphylococcus aureus* colonization in veterinary personnel attending an international equine veterinary conference. Vet. Microbiol..

[6] Fahey J.W., Zalcmann A.T., Talalay P. (2001). The chemical diversity and distribution of glucosinolates and isothiocyanates among plants. Phytochemistry.

[7] Vaughn S.F., Berhow M.A. (2005). Glucosinolate hydrolysis products from various plant sources: pH effects, isolation, and purification. Ind. Crops Prod..

[8] Thejass P., Kuttan G. (2007). Allyl isothiocyanate (AITC) and phenyl isothiocyanate (PITC) inhibit tumour-specific angiogenesis by downregulating nitric oxide (NO) and tumour necrosis factor-alpha (TNF-alpha) production. Nitric. Oxide-Biol. Chem..

[9] Qazi A., Pal J., Maitah M., Fulciniti M., Pelluru D., Nanjappa P., Lee S., Batchu R.B., Prasad M., Bryant C.S. (2010). Anticancer activity of a broccoli derivative, sulforaphane, in Barrett adenocarcinoma: Potential use in hemoprevention and as adjuvant in chemotherapy. Transl. Oncol..

[10] O’Callaghan K.J., Stone P.J., Hu X., Griffiths D.W., Davey M.R., Cocking E.C. (2000). Effects of glucosinolates and flavonoids on colonization of the roost of *Brassica napus* by *Azorhizobium caulinodans* ORS571. Appl. Environ. Microbiol..

[11] Hong E.Y., Kim G.H. (2008). Anticancer and antibacterial activities of β-phenylethyl isothiocyanate in *Brassica rapa* L.. Food Sci. Technol. Res..

[12] Luciano F.B., Holley R.A. (2009). Enzymatic inhibition by allyl isothiocyanate and factors affecting its antimicrobial action against *Escherichia coli* O157:H7. Int. J. Food Microbiol..

[13] Kim M.G., Lee H.S. (2009). Growth-inhibiting activities of phenethylisothiocyanate and its derivatives against intestinal bacteria. J. Food Sci..

[14] Wilson A.E., Bergaentzlé M., Bindler F., Marchioni E. (2013). *In vitro* efficacies of various isothiocyanates from cruciferous vegetables as antimicrobial agents against foodborne pathogens and spoilage bacteria. Food Control.

[15] Troncoso R., Espinoza C., Sánchez-Estrada A., Tiznado M.E., García H.S. (2005). Analysis of the isothiocyanates present in cabbage leaves extract and their potential application to control *Alternaria* rot in bell peppers. Food Res. Int..

[16] Verkerk R., Schreiner M., Krumbein A., Ciska E., Holst B., Rowland I. (2009). Glucosinolates in Brassica vegetables; the influence of the food supply chain on intake; bioavailability and human health. Mol. Nutr. Food Res..

[17] Herzallah S., Holley R. (2012). Determination of sinigrin, sinalbin, allyl- and benzyl isothiocyanates by RP-HPLC in mustard powder extracts. LWT-Food Sci. Technol..

[18] Vermeulen M., van Den Berg R., Freidig A.P., van Bladeren P.J., Vaes W.H.J. (2006). Association between consumption of cruciferous vegetables and condiments and excretion in urine of isothiocyanate mercapturic acids. J. Agric. Food Chem..

[19] Matthäus B., Fiebig H.J. (1996). Simultaneous determination of isothiocyanates, indoles, and oxazolidinethiones in myrosinase digests of rapeseeds and rapeseed meal by HPLC. J. Agric. Food Chem..

[20] Desbois A.P., Gemmell C.G., Coote P.J. (2010). *In vivo* efficacy of the antimicrobial peptide ranalexin in combination with the endopeptidase lysostaphin against wound and systemic meticillin-resistant *Staphylococcus aureus* (MRSA) infections. Int. J. Antimicrob. Agents.

[21] Jang M., Hong E.Y., Kim G.H. (2010). Evaluation of antibacterial activity of 3-butenyl, 4-pentenyl, 2-phenylethyl, and benzyl isothiocyanate in Brassica vegetables. J. Food Sci..

[22] Bal A.M., Garau J., Gould I.M., Liao C.H., Mazzei T. (2013). Vancomycin in the treatment of meticillin-resistant *Staphylococcus aureus* (MRSA) infection, End of an era?. J. Glob. Antibiot. Res..

[23] Jacob C., Anwar A. (2008). The chemistry behind redox regulation with a focus on sulphur redox systems. Physiol. Plant..

[24] Shapiro T.A., Fahey J.W., Wade K.L., Stephenson K.K., Talalay P. (1998). Human metabolism and excretion of cancer chemoprotective glucosinolates and isothiocyanates of cruciferous vegetables. Cancer Epidemiol. Biomark. Prev..

[25] Kassie F., Pool-Zobel B., Parzefall W., Knasmuller S. (1999). Genotoxic effects of benzyl isothiocyanate, a natural chemopreventive agent. Mutagenesis.

[26] Cockerill F.R., Wikle M.A., Alder J., Dudley M.N., Eliopoulos G.M., Ferrar M.J., Hardy D.J., Hecht D.W., Hindl J.A., Patel J.B. (2012). Methods for Dilution Antimicrobial Susceptibility Tests for Bacteria that Grow Aerobically.

[27] Sarker S.D., Nahar L., Kumarasamyn Y. (2007). Microtitre plate-based antibacterial assay incorporating resazurin as an indicator of cell growth; and its application in the *in vitro*** antibacterial screening of phytochemicals. Methods.

